# TB stigma in India: A narrative review of types of stigma, gender differences, and potential interventions

**DOI:** 10.1371/journal.pgph.0005109

**Published:** 2025-09-22

**Authors:** Madeline E. Carwile, Senbagavalli Prakash Babu, Chelsie Cintron, Madolyn Dauphinais, Susie Jiaxing Pan, Mahalakshmi Thulasingam, C. Robert Horsburgh, Sonali Sarkar, Natasha S. Hochberg, Lora L. Sabin, David Flynn, Blessina Kumar, Pranay Sinha, Urvashi B. Singh

**Affiliations:** 1 Boston Medical Center, Boston, Massachusetts, United States of America; 2 Department of Preventive and Social Medicine, Jawaharlal Institute of Postgraduate Medical Education and Research, Puducherry, India; 3 College of Arts and Sciences, Boston University, Boston, Massachusetts, United States of America; 4 Department of Global Health, Boston University School of Public Health, Boston, Massachusetts, United States of America; 5 Section of Infectious Diseases, Department of Medicine, Boston University School of Medicine, Boston, Massachusetts, United States of America; 6 Department of Medical Sciences & Education, Boston University School of Medicine, Boston, Massachusetts, United States of America; 7 Global Coalition of TB Advocates, New Delhi, India; 8 All India Institute of Medical Sciences, New Delhi, India; York University, CANADA

## Abstract

In India, persons with tuberculosis (PWTB) and their households experience significant disease-related stigma. The objective of this narrative review was to conduct a review of existing literature related to the types of stigma experienced by PWTB and their household members, with a focus on the effects of stigma, possible interventions, and gender differences. A literature search was conducted on PubMed, EMBASE, and Web of Science using key search terms. We found that tuberculosis (TB)-related stigma has negative effects on emotional and mental health, relationships, and treatment adherence. Women experience a higher burden of TB stigma compared to men. Moreover, TB stigma can affect mental well-being and lead directly to reductions in the number of PTWB seeking treatment, treatment adherence, and treatment completion. All these factors can lead to negative health outcomes for the PWTB, higher costs to the government, and even the spread of the infectious disease to other members of the community. The consequences of TB-related stigma require additional attention.

## Introduction

Tuberculosis (TB) has regained its status as the leading infectious killer worldwide after briefly ceding that position to COVID-19 [1]. India shoulders a quarter of the global burden of TB, with an estimated incidence of 2.82 million cases and 342,000 deaths in 2022 [2]. TB inflicts severe economic stress on persons with TB (PWTB). Eliminating catastrophic costs is a key End-TB Strategy goal, and the economic impacts of TB have been well studied [3], but less focus has been given to the social impacts of TB, including stigma [4]. TB stigma continues to be a significant problem in India. One study from Chennai showed that an estimated 69% of PWTB experienced a social impact from TB, compared to the 30% that experienced an economic impact (with 24% experiencing both) [4]. Indeed, in a 2004 study examining stigma indices in four countries using a quantitative approach, India was found to have the highest stigma index (1.17 compared to 0.99, 0.85, and 1.03 in Bangladesh, Malawi, and Colombia, respectively) [5].

Stigma is often at the root of social impacts such as difficulties with family, work, and education. Stigma is the labeling of an individual with an undesirable stereotype, which results in discrimination and social rejection [6]. Stigma includes discrimination against those that are seen as different from others, and individuals who are concerned about experiencing stigma may choose to hide their stigmatized identity or otherwise minimize the negative consequences [7]. As such, higher levels of stigma may increase the barriers to detection and decrease treatment adherence [8–11]. While other studies of TB stigma in India have focused on one particular geographic area or one dimension of stigma, we did not find reviews that addressed diverse regions and different types of stigma. This narrative review aims to describe the effects of TB-related stigma across India, focusing primarily on the disparate experiences of men and women with TB.

## Methods

We conducted a literature search using PubMed, EMBASE, and Web of Science using the following key search terms: ‘Tuberculosis,’ ‘social impact,’ ‘gender,’ ‘India,’ and ‘stigma.’ Within PubMed, we used more targeted MeSH terms to improve the accuracy and completeness of the results (see S1 Text). We reviewed the titles and abstracts of the first 200 search results to find relevant terms. We included all studies up to May 19, 2025 in the search parameters, with no restriction on the start date. We found additional references by reviewing the bibliographies of the articles found through database search.

Initial screening of abstracts and articles was conducted by a primary reviewer, assisted by a student assistant. Selected articles were subsequently cross-checked independently by a second reviewer to ensure reliability. After resolving duplicate results and excluding studies where stigma was not a main focus, we identified 44 relevant studies related to the effects of TB-related stigma in India. The papers we present in this narrative review used both quantitative and qualitative methods (that involved in-depth interviews with pre-coded themes, with several using the semi-structured Explanatory Model Interview Catalogue [EMIC], which allows for quantifying stigma using validated indices) [12]. Additional studies were also used in our analysis to examine potential interventions for TB-related stigma.

Disease-related stigma, including TB stigma, is often categorized into four types: anticipated stigma, perceived community stigma, enacted stigma, and internalized stigma, although there are some differences in how these categories are defined, particularly the definition of perceived stigma [13]. While some studies use ‘perceived stigma’ to refer to self-perceived stigma by PWTB, in this review we focused on perceived community stigma. We defined the terms as follows. *Anticipated stigma* occurs when a person believes that they will be stereotyped or discriminated against by others [6]. *Perceived community stigma* refers to those without the disease treating those affected by the disease differently or considering them to be different [13]. *Enacted stigma* refers to specific, actual experiences of discrimination or negative treatment [14]. *Internalized stigma* occurs when individuals endorse negative stereotypes and apply them to themselves [13].

## Results

**Fig 1:**
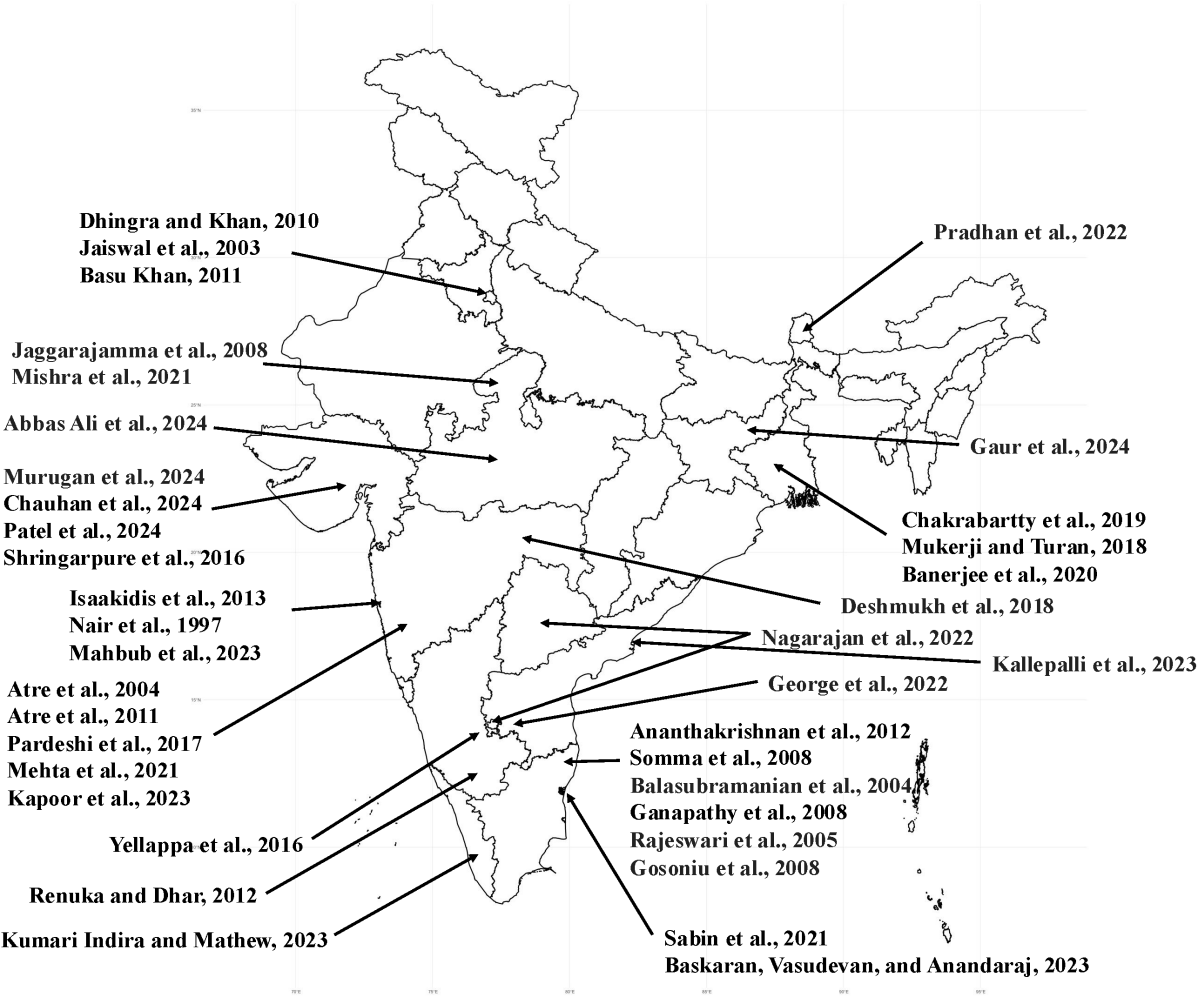
The location of the studies included in the review. Review papers and those using data from across India are not included in this map.

**Fig 2:**
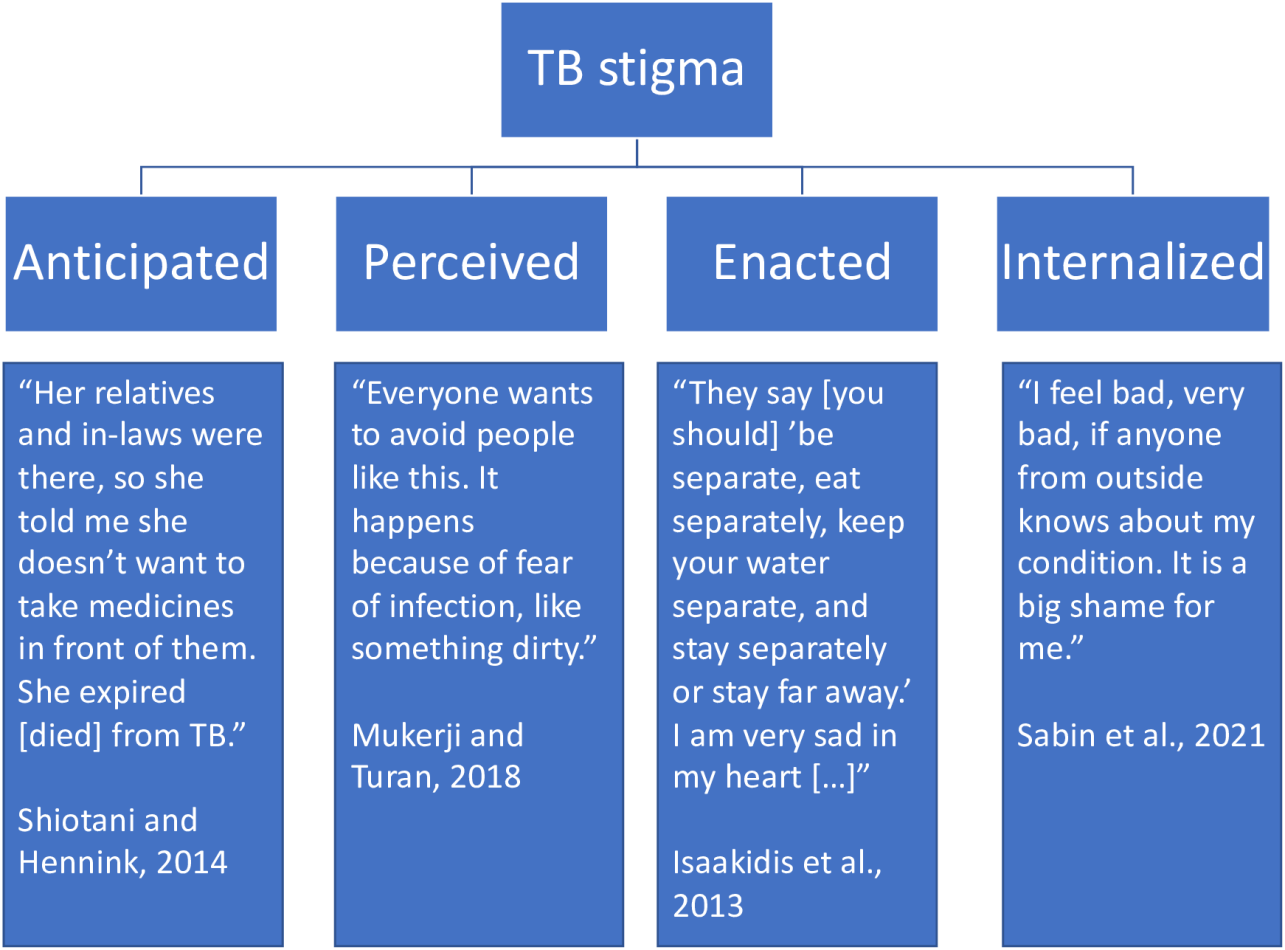
Examples of the four principal types of stigma: anticipated, perceived, enacted, and internalized.

Through our search strategy, we identified 44 papers, which are summarized in Table 1. A map of the study locations can be found in Fig 1. Fig 2 displays examples of the four types of stigma from Indian study participants.

**Table 1:** A summary of the studies included in this narrative review.

Study name	Location	Participants	Key findings
Gender differentials in tuberculosis: the role of socio-economic and cultural factors (Hudelson, 1996)	n/a	n/a	• When a household member becomes ill, women are more likely to care for the PWTB and to take on a greater burden of household activities • Women experience more disease-related stigma than men, and these effects extend to their family and children
Tuberculosis in Bombay: new insights from poor urban patients (Nair, George, and Chacko, 1997)	Mumbai	16 PWTB from a total of 120 who attended the PATH clinic in Mumbai	• Women were particularly vulnerable to dismissal from work as domestic workers if their employers learned of their TB status • Married female PWTB received less family support than married men or unmarried women • Men primarily reported concern about loss of wages and inability to work, while women primarily reported concern about rejection from their husband and in-laws and reduced marriage prospects • Women had increased pressure to keep their disease secret, as well as increased difficulties in doing so due to closer monitoring of their movements
Adherence to tuberculosis treatment: lessons from the urban setting of Delhi, India (Jaiswal et al., 2003)	Two chest clinics in Delhi	40 PWTB who left TB treatment before treatment was completed	• 6.5% of PWTB at one clinic and 10% of PWTB at another left treatment before completion • Rude behavior from healthcare workers was one factor that led to incomplete treatment
Cultural concepts of tuberculosis and gender among the general population without tuberculosis in rural Maharashtra, India (Atre et al., 2004)	Pune District, Maharashtra	80 men and 80 women who did not have TB and whose immediate family members did not have TB	• Respondents stated that women were more likely to hide their TB compared to men, and that this was due to greater social stigma • Participants reported that they would not want their son to marry a woman who had TB, and would expect her to be sent back home if TB developed after the marriage • Respondents often had incorrect beliefs on the transmission of TB, which could increase stigma
Gender disparities in tuberculosis: report from a rural DOTS programme in south India (Balasubramanian et al., 2004)	Thiruvallur District	2,115 PWTB (following a community survey of over 76,000 adults and a review of PWTB enrolled in TB treatment)	• Female PWTB were significantly more likely to experience stigma or be excluded from social events compared to male PWTB • 21% of female PWTB and 14% of male PWTB felt inhibited discussing their illness with friends or family (P < 0.05)
Perceptions of tuberculosis patients about their physical, mental and social well-being: a field report from south India (Rajeswari et al., 2005)	Two tuberculosis units (TUs) in Tiruvallur district of Tamil Nadu	610 PWTB registered for treatment between July and December 2000	• PWTB felt unable to visit friends or tell friends and family about their diagnosis • While quality of life improved following TB treatment, stigma continued even after treatment was completed
Gender and socio-cultural determinants of TB-related stigma in Bangladesh, India, Malawi and Colombia (Somma et al., 2008)	10 tuberculosis treatment centers in Chennai	Approximately 100 PWTB from Chennai (and around 100 each from Bangladesh, Malawi, and Colombia)	• The researchers identified 18 categories of TB-related stigma • India had the highest stigma index of the four countries (1.17 compared to 0.99, 0.85, and 1.03) • Females experienced a higher rate of stigma (1.28) compared to males (1.08) (p = 0.11) • The highest category of stigma for PWTB in India was ‘desire to keep others from knowing’
Gender and socio-cultural determinants of delay to diagnosis of TB in Bangladesh, India and Malawi (Gosoniu et al., 2008)	TB Control Programs in Bangladesh, India, and Malawi. The India site was comprised of TB Units in Chennai.	127 PWTB in India (102 in Bangladesh and 100 in Malawi)	• Female sex and being a married woman were associated with diagnosis delay in India • Married female PWTB described neglect and poor treatment from their husbands and in-laws • Female PWTB also described keeping away from others to avoid spreading TB, fearing social stigma, and non-disclosure of their TB status, even to husbands or other family members
Perceptions of gender and tuberculosis in a south Indian urban community (Ganapathy et al., 2008)	Chennai	Four focus groups each consisting of 8–10 adult community members	• Nearly three times more men are detected by the RNTCP compared to women, and understanding community beliefs could help explain why • Participants reported that men would receive more family support in seeking care, and would be more likely to buy costly medication and see private doctors • Respondents stated that male PWTB could get married, but that it would be difficult for female PWTB
Psycho-social dysfunction: perceived and enacted stigma among tuberculosis patients registered under revised national tuberculosis control programme (Jaggarajamma et al., 2008)	A rural area near the village of Saharana in Madhya Pradesh	276 PWTB registered for treatment during January-March 2004 in government health facilities	• Male and female participants reported similar levels of ‘social’ (enacted) stigma • Both genders reported higher levels of perceived stigma, with males describing higher levels than females
A sociological study on stigma among TB patients in Delhi (Dhingra and Khan, 2010)	New Delhi	PWTB receiving treatment under the RNTCP	• 60% of PWTB reported hiding their diseases status from friends and neighbors (p < 0.05) • Female PWTB reported higher levels of stigma compared to male PWTB
Gender and community views of stigma and tuberculosis in rural Maharashtra, India (Atre et al., 2011)	Western Maharashtra	160 respondents without TB from 20 randomly selected villages in Western Maharashtra	• Researchers classified seven aspects of stigma • For both the female and male vignettes, the highest mean prominence was for the indicators ‘keep others from knowing’ and ‘think less of self’ • Respondents reported a desire to stay away from PWTB and keep their away from them, as well as a need to isolate the PWTB and their belongings • Respondents also reported that there would be difficulties in arranging marriages, both for the PWTB and their family members, as well as difficulties in ongoing marriages
Understanding the Gender Aspects of Tuberculosis: A Narrative Analysis of the Lived Experiences of Women With TB in Slums of Delhi, India (Basu Khan, 2011)	Two slums in Delhi	PWTB, and men and women without TB	• Participants connected developing TB with having violated social norms, such as alcohol and drug use and sexual activity • Community members stigmatized PWTB, and the stigma was stronger for female PWTB • PWTB tried to hide their disease status, or avoided treatment • Participants suggested that women with TB would have a hard time finding a husband
Socioeconomic impact of TB on patients registered within RNTCP and their families in the year 2007 in Chennai, India (Ananthakrishnan et al., 2012)	Chennai	300 PWTB	• 69% of PWTB experienced a social impact from TB and 30.3% experienced an economic impact (with 75% experiencing at least one and 24.3% experiencing both) • Women experienced a greater social impact than men, with 80.7% of women and 61.8% of men reporting social impacts from TB (p-value = 0.001) • Major forms of social impacts included fear of rejection (38%), fear of discrimination (31%), having to depend on others (20%), and not telling family members about disease status (14%)
‘I cry every day’: experiences of patients co-infected with HIV and multidrug-resistant tuberculosis (Isaakidis et al., 2013)	Mumbai	12 HIV/MDR-TB co-infected individuals receiving treatment in an MSF clinic	• PWTB-HIV described non-disclosure of their disease status, as well as exclusion and loss of support after revealing their diseases • Support from healthcare workers and family members was associated with increased adherence to treatment and reduced stress
Knowledge and awareness of tuberculosis among high school students of Mysore City (Renuka and Dhar, 2013)	Mysore	129 high school students	• The high school students surveyed had an overall high level of TB-related knowledge • 35% of high school students said that PWTB should not get married
Socio-cultural influences on adherence to tuberculosis treatment in rural India (Shiotani and Hennink, 2014)	Rural India	PWTB and healthcare providers	• Cultural norms and social contexts, including gender role, can affect a PWTB’s adherence to treatment • Women are more likely than men to experience TB-related stigma, including difficulty with marriage prospects • PWTB experienced depression and anxiety due to their TB status, which can reduce treatment adherence
Patient and provider reported reasons for lost to Follow Up in MDRTB Treatment: A Qualitative Study from a Drug Resistant TB Centre in India (Deshmukh et al., 2015)	Seven districts of eastern Maharashtra	20 persons with MDR-TB who were lost to follow up, and 10 providers	• PWTB reported poor treatment and perceived lack of caring by healthcare providers • PWTB described strategies that they took to avoid disclosure of their disease status, including avoiding treatment visits • Participants described additional social stigma for women, including difficulties arranging marriages and problems in existing marriages
Coping with tuberculosis and directly observed treatment: a qualitative study among patients from South India (Yellappa et al., 2016)	Tumkur district, Karnataka	33 PWTB, with three different categories of treatment location/method of diagnosis	• Some PWTB reported that they would stop taking treatment rather than risk losing their job or work hours. Family members also had their work disrupted by taking PWTB to receive DOTS treatment • Social stigma can impact a PWTB’s future marriage prospects • PWTB particularly younger ones, reported hiding their disease. PWTB in rural areas reported higher levels of difficulty in hiding their disease compared to those in urban areas • Many PWTB reported ‘self-induced isolation’ in which they did not want to share utensils or allow children to come near them
“When Treatment Is More Challenging than the Disease”: A Qualitative Study of MDR-TB Patient Retention (Shringarpure et al., 2016)	Gujarat	PWTB at a DR-TB treatment site who were lost to follow-up, as well as DOT providers and supervisors	• Stigma, including unfriendly healthcare providers, was a major cause of loss to follow-up among PWTB • Education and counseling could improve treatment adherence
Is Knowledge Regarding Tuberculosis Associated with Stigmatising and Discriminating Attitudes of General Population towards Tuberculosis Patients? Findings from a Community Based Survey in 30 Districts of India (Sagili, Satyanarayana, and Chadha, 2016)	30 districts of India	4,562 respondents from general population (3,823 were included in the analysis)	• 17% of respondents had ‘appropriate knowledge about TB’ (95% CI 15.6–18.0) • Respondents in urban (vs. rural) areas and from middle-high income groups (vs. low income) were twice as likely to have appropriate TB knowledge • 73% of respondents (95% CI 71.4–74.2) showed a stigmatizing attitude, and 98% a discriminating attitude (95% CI 97.4–98.3) • Respondents agreed with statements such as refusing to share a meal with a PWTB or not marrying their son or daughter to a PWTB • Stigmatizing and discriminating attitudes were both independent of level of TB knowledge
Resident doctors’ attitudes toward tuberculosis patients (Pardeshi et al., 2017)	B.J. Government Medical College and Sassoon General Hospital, Pune	212 postgraduate resident medical doctors, 132 of whom see PWTB daily	• 49% of the physicians reported experiencing compassion and a desire to help PWTB • Physicians who attended a training program were three times more likely to report compassion and a desire to help compared to physicians who did not attend training (p = 0.005; OR = 2.95, 95% CI) • Physicians who knew a colleague with TB were significantly less likely to avoid PWTB (p = 0.002; OR = 2.82, 95% CI)
Exploring Manifestations of TB-Related Stigma Experienced by Women in Kolkata, India (Mukerji and Turan, 2018)	Kolkata	Twenty female PWTB either currently undergoing TB treatment or who had completed treatment	• Community members regularly avoided PWTB, and some healthcare workers also kept their distance from PWTB • PWTB reported high levels of gossip related to their TB status, and stated that women experienced more gossip than men • Participants described having engagements called off, and fear of exclusion from work or school prompted PWTB to keep their disease status hidden • Female participants reported a lack of support from their husbands and family members, including still having to complete household chores • PWTB described feeling guilt, low self-worth, and feeling that they would be better off dead
Tuberculosis related stigma attached to the adherence of Directly Observed Treatment Short Course (DOTS) in West Bengal, India (Chakrabartty et al., 2019)	Three districts in West Bengal	145 people who did not complete TB treatment. 51 were in the low stigma group and 94 were in the high stigma group.	Those in low stigma group were 8.59 times more likely to have late non-completion of treatment than those in the high stigma group (p = 0.001)
Self-reported tuberculosis in India: evidence from NFHS-4 (Mazumdar, Satyanarayana, and Pai, 2019)	India	A nationally representative sample of over 600 000 households comprising of about 2.9 million individuals	• TB knowledge and awareness was relatively high, but lower among illiterate and lower-educated respondents • 13.8% of women and 17.5% of men would keep it a secret if a family member had TB
Perceived Discrimination among Tuberculosis Patients in an Urban Area of Kolkata City, India (Banerjee et al., 2020)	Kolkata	140 PWTB receiving treatment at DOTS centers.	• Disclosure of disease status was higher to family members (98.5%) than to neighbors and colleagues (66.4%, and 62.1%, respectively) • Perceived discrimination was a predictor of unsuccessful treatment (AOR [95% CI] = 2.61 [1.04–7.84])
Tuberculosis and Stigma in India: Evidence from a Nationally Representive Survey (Barman, 2020)	India	Adult men and women who participated in the NFHS-3	• 18.6% of men and 19.4% of women would keep secret a family member’s TB diagnosis
Reasons for loss to follow-up (LTFU) of pulmonary TB (PTB) patients: A qualitative study among Saharia, a particularly vulnerable tribal group of Madhya Pradesh, India (Mishra et al., 2021)	A rural area near the village of Saharana in Madhya Pradesh	22 PWTB, 10 treatment providers, and 10 family members of PWTB	• PWTB reported that they did not want to visit the DOT center, or for HCWs to visit their homes, due to fear of disclosure of their TB status • Fear of social stigma was a barrier to treatment and a contributor to loss to follow up
“People listen more to what actors say”: A qualitative study of tuberculosis-related knowledge, behaviours, stigma, and potential interventions in Puducherry, India (Sabin et al., 2021)	Puducherry	47 PWTB and household members (in-depth interviews) and PWTB, household members, and key stakeholders (eight focus group discussions)	• Most PWTB reported hiding their disease status from friends and neighbors, expressing fears of stigma or poor treatment • Participants suggested that women experienced more stigma than men, and suggested that female PWTB and female family members would have problems arranging marriages • Almost half of participants reported experiencing enacted stigma, including poor treatment, verbal abuse, negative experiences with healthcare workers, and losing their jobs • PWTB shared their feelings of guilt and shame, including thoughts of suicide • Participants suggested many different types of interventions to reduce stigma, with the most popular including celebrity spokespersons, social media, school-based education, support groups, and counseling
TB-related knowledge and stigma among pregnant women in low-resource settings (Mehta et al., 2021)	Urban Pune	Study conducted among 202 pregnant women with and without LTBI.	• Participants generally had a high level of TB knowledge • 40% stated it was shameful to have TB, and 29% stated that they would hide their diagnosis from others if they developed TB • Limited knowledge about TB was significantly associated with stigmatizing attitudes (odds ratio 14.99, 95% confidence interval 6.98–32.1)
Internalized and perceived stigma and depression in pulmonary tuberculosis: do they explain the relationship between drug sensitivity status and adherence? (Pradhan et al., 2022)	Sikkim	71 PWTB who were on drug-sensitive TB regimen	• Persons with higher internalized and perceived stigma are more likely to develop depression, which negatively affects adherence • Persons on the MDR-TB regimen have higher stigma compared to those on a standard TB regimen
A dual perspective of psycho-social barriers and challenges experienced by drug-resistant TB patients and their caregivers through the course of diagnosis and treatment: findings from a qualitative study in Bengaluru and Hyderabad districts of South India (Nagarajan et al., 2022)	Bengaluru and Hyderabad	PWTB who had completed DR-TB treatment (n = 20) and caregivers (n = 20)	• Persons with DR-TB and their caregivers experienced considerable emotional and social consequences due to TB • Fear of disease discovery led some PWTB to leave their jobs or school • Enacted stigma and discrimination occurred during the diagnosis and treatment stages
Barriers to treatment adherence for female Tuberculosis (TB) patients during the COVID-19 pandemic: Qualitative evidence from front-line TB interventions in Bengaluru City, India (George et al., 2022)	Bengaluru	188 female PWTB who underwent DOTS treatment	• Some families stigmatized women when they were diagnosed with TB • PWTB feared losing employment if the disease was disclosed to their employer, and also feared stigmatization by family members, relatives, friends, and neighbors
“One-by-One, TB Took Everything Away From Me”: A Photovoice Exploration of Stigma in Women with Drug-Resistant Tuberculosis in Mumbai (Mahbub et al., 2023)	Mumbai	Nine women with drug-resistant TB attending a clinic in Mumbai	• Participants experienced enacted stigma from both family members and healthcare providers, including being reprimanded and being moved to the end of the line or outside of a waiting room • Participants also reported anticipated stigma, and engaged in actions to hide either their disease status or themselves • PWTB experienced low self-worth and other forms of internalized stigma • Positive thinking and individual resilience could help reduce the effects of stigma • Connecting with other PWTB could help increase collective resilience
Prevalence of stigma among TB patients and its associated factors – a community based cross-sectional study in Puducherry, India (Baskaran, Vasudevan, and Anandaraj, 2023)	Puducherry	420 adult drug sensitive non-HIV PWTB registered under the NTEP in Puducherry district	• The prevalence of stigma among PWTB was 69.3% • Perceived stigma was noted in 47.1% of participants, while 33.6% had self-stigma and 26.0% had experienced stigma • On measuring the impact of stigma, 52.6% reported participation restriction • Illiteracy and lower-socio economic status were found to be significantly associated with TB stigma
Burden of stigma among tuberculosis patients: a cross-sectional study (Kallepalli et al., 2023)	Kakinada District, Andhra Pradesh	100 PWTB above 18 years of age	• A majority of PWTB (59%) reported perceived stigma • 73% of PWTB revealed that they wanted to keep others from knowing about their condition • 46% of PWTB felt shame or embarrassment due to their diagnosis • PWTB with lower knowledge of the disease were more likely to experience stigma, and those who experienced stigma were less likely to visit the TB clinic (p < 0.05)
TB related stigma and gender disparity among unaffected population in central Kerala, a survey (Kumari Indira and Mathew, 2023)	Ernakulam district of Kerala	Persons without TB visiting the out-patient department of a tertiary care center	• Overall stigma scoring was low (mean score = 15.9; total 75) • Community stigma was higher among females compared with males (p < 0.002), particularly among females receiving female vignettes (chi-square = 14.1, p < 0.0001). The association was significant even after adjusting for co-variables (OR = 3.323, p = 0.005)
Depression among tuberculosis patients and its socio-demographic correlates: a cross-sectional study from Western Maharashtra (Kapoor et al., 2023)	Western Maharashtra	Adult PWTB taking treatment at DOTS centers who have completed at least 1 month of treatment	• There was a significant association between perceived stigma and depression (P = 0.001) • Odds of depression among participants with perceived stigma was 15.24 times in comparison to the odds of depression in participants with no perceived stigma
A cross-sectional study to assess stigma associated with tuberculosis in patients, family members, and health care staff in central India (Abbas Ali et al., 2024)	Bhopal district, Madhya Pradesh	314 participants stratified into three groups: PWTB, family members, and healthcare workers	• Among all 314 participants, the prevalence of stigma was 26.75% • A statistically significant correlation was found between stigma experienced and marital status (p = 0.013) and level of knowledge regarding tuberculosis (p < 0.001) • Among PWTB, the odds of stigma were 13.25 (C.I. 95%: 4.14, 42.41) times higher in females and 3 (C.I. 95%: 1.005, 8.95) times higher in persons with unsatisfactory knowledge about tuberculosis compared to males and persons with limited knowledge, respectively
Mental Health Impacts of Multidrug-Resistant Tuberculosis in Patients and Household Contacts: A Mixed Methods Study (Murugan et al, 2024)	Gujarat	403 persons with MDR-TB and 403 household contacts	• Persons with MDR-TB were significantly more likely than household contacts to have depression and anxiety • Female gender (AOR 2.5, 95% CI 1.1-6.0) and perceived stigma (AOR 3.2, 95% CI 1.1-5.3) were associated with depression • Perceived stigma (AOR 2.2, 95% CI 1.1-6.3) was associated with anxiety
Dual burden of TB and mental ill-health: Prevalence and associated factors of anxiety and depression among TB patients in Gujarat (Chauhan et al., 2024)	Gujarat (Gandhinagar and Surat)	PWTB from Gujarat	• PWTB with high perceived stigma and who had been sick for over six months were 3.1 times more likely to experience anxiety symptoms (AOR 3.10, 95% CI: 2.22, 4.23) and 1.6 times more likely to experience symptoms of depression (AOR 1.60, 95% CI: 1.12, 2.53). • Gender, occupation, and socioeconomic level were significantly associated with both anxiety and depression (P < 0.05). • Being single (AOR: 3.29; 95% CI: 2.45-7.53), low socioeconomic status (AOR: 5.41; 95% CI: 2.44-7.97), and being on TB treatment (AOR: 4.35; 95% CI: 1.83-15.65) were strongly associated with anxiety and depressive symptoms.
Knowledge, attitude and perceived barriers related to directly observed treatment, short-course among patients and caregivers attending tuberculosis clinics: a cross-sectional survey (Gaur et al., 2024)	Deoghar, Jharkhand	180 PWTB and 217 caregivers at selected TB clinics	Distance to treatment facilities (95.6%), the necessity to take time off from work (91.7%), and social stigma (65.0%) were identified as the top three barriers to DOTS adherence.
The Invisible Toll: Unveiling the Prevalence and Predictors of Depression and Anxiety Among Pulmonary Tuberculosis (TB) Patients and Their Households in Gujarat, India (Patel et al., 2024)	Jamnagar, Gujarat, India	272 PWTB and 544 household contacts at TB units	• Depression and anxiety are highly prevalent among persons with pulmonary TB and their household contacts • Low socioeconomic status, lack of social support, and TB-related stigma are significant predictors of mental health conditions • Integrated, multidisciplinary interventions are needed to address the psychological impact of TB
Evaluation of health-related quality of life and adherence among pre-extensively drug-resistant tuberculosis patients receiving either Bedaquiline or Delamanid regimen at a teaching hospital in Eastern India (Paikray et al, 2024)	Eastern India	85 PWTB of both genders aged 6–18 years for the Delamanid group, and above 18 years for both Bedaquiline and Delamanid groups	• High stigma levels, new diagnosis with XDR-TB, and experiencing more adverse events were all associated with higher nonadherence to treatment • Individuals reporting high stigma had 20.27 times higher odds of nonadherence

### Anticipated stigma

Anticipated stigma most commonly manifests in a desire to hide one’s own disease status or the disease status of family members [15–22]. A 2010 study of 1,977 PWTB found that 60% of the respondents hid their disease status from neighbors and friends [15]. In a 2021 study of pregnant women in Pune, 29% stated that they would hide their disease status if they were to develop TB [23], while another study found that 13.8% of women and 17.5% of men would be secretive about the TB diagnosis of a family member [18]. Similarly, a 2020 study found that 18.6% of men and 19.4% of women would hide the TB status of a family member [24]. A 2023 study found that 73% of PWTB wanted to keep others from knowing about their TB status [25]. This desire to hide one’s disease status was associated with fears of rejection and discrimination, which were reported by 38% and 31% of respondents, respectively, in data collected in 2007 in Chennai [4]. In other studies, PWTB revealed fear of being excluded from work or school if they revealed their TB diagnosis, or if someone discovered their TB status based on their symptoms [13,20,26].

The desire to hide disease status from family members was less common [4,15,27]. In a 2020 study of PWTB in Kolkata, 98.5% of PWTB disclosed their disease status to family, compared to 66.4% to neighbors and 62.1% to colleagues [28]. In a study using 33 in-depth interviews of PWTB in Karnataka, younger PWTB were more likely to hide their disease compared to older PWTB [27]. Persons in rural areas had less success than those in urban areas due to increased interactions with neighbors [27].

### Perceived community stigma

A study of over 4,000 members of the general population in 30 districts of India found that 73% and 98% of respondents, respectively, had stigmatizing and discriminating views towards PWTB [29]. Community members stated that PWTB were a threat to the community and that they would not share a meal with a PWTB [29]. In a study in Delhi, community members associated developing TB with engaging in “deviant” behaviors, such as using drugs or alcohol or engaging in indiscriminate sexual activity [22].

Researchers interviewed 80 male and 80 female community members in Maharashtra after presenting them with vignettes describing PWTB [30]. The respondents described a desire to stay away from PWTB and their children and felt that PWTB and their belongings should be isolated [30]. Perceived stigma also intersects with anticipated stigma, with community respondents stating that other people would be likely to stigmatize PWTB. For instance, one respondent explained that:

*People will not talk to her. They will not go near her or eat food cooked by her. Her family members will keep her utensils separate. Even while offering tea, she will clean the utensils with hot water* [30].

Perceived stigma has important implications for marital prospects of PWTB and their family members. Respondents stated that arranging marriages for PWTB and their family members would be challenging and there would also be difficulties in current marriages, such as marital discord or a lack of support [30]. In a survey in Mysore in 2012, 35% of high school students said that PWTB should not get married [31].

A 2014 survey on 212 physicians in Pune found that only 49% of the physicians reported compassion and a desire to help PWTB [32]. Stigma from physicians has serious implications, including lower treatment completion rates among PWTB who felt unsupported during their treatment [27,32,33]. Importantly, physicians who attended a training program or knew a colleague with TB were three times more likely to report compassion and a desire to help compared to physicians who did not do so [32].

### Enacted stigma

Enacted stigma could come from community members, family members, and healthcare workers. While experiences of enacted stigma were less common than anticipated and perceived stigma, enacted stigma was substantially higher in women than in men. For instance, women experienced more community gossip and verbal abuse than men [13]. Moreover, women reported engagements being called off after their TB diagnosis [13]. Married women mentioned abandonment and isolation from both their husbands and other family members after receiving their TB diagnosis [34]. PWTB and their household members also described not being allowed to use common areas or bathrooms, relatives refusing to eat or drink at their house, losing their jobs, and having their neighbors prevent their children from playing together [19,21].

Healthcare workers were also sources of enacted stigma. In a 2003 study of PWTB who left treatment before completion, PWTB cited rude behavior from healthcare workers as a reason for discontinuing treatment [33], as did PWTB in a 2016 study in Gujarat [35]. In a 2015 qualitative study of PWTB who were lost to follow-up, PWTB reported poor treatment from their healthcare providers [36], while a qualitative study from 2021 found that nearly a quarter of PWTB interviewed had experienced negative treatment from their healthcare providers [19]. In a qualitative photovoice study from 2023, a participant shared a story of being shouted at and forced to wait until all non-TB patients had left before she would be seen by healthcare providers [21]. Other participants shared similar stories of being reprimanded or moved to the end of the waiting room line.

### Internalized stigma

PWTB reported decreased self-worth due to their TB status [13,21], as well as feelings of shame and embarrassment [25]. In a study of 202 pregnant women with and without latent TB, 40% stated that it was shameful to have TB [23].

PWTB also described feelings of anxiety and depression, as well as suicidal ideation due to stigma [17,19,37,38]. A 2024 study found that 37.5% of persons with MDR-TB experienced depression, while 45.2% experienced anxiety, significantly higher than their household contacts [37]. Stigma was associated with three-fold higher odds of depression and two-fold higher odds of anxiety among PWTB [37]. Persons with higher internalized and perceived stigma are more likely to develop depression, which negatively affects adherence [39].

### Gender differences in stigma

The majority of studies showed that women experience disproportionately greater TB stigma. One study found that the odds of stigma were 13.25 (95% CI 4.14-42.1) times higher in women compared to men [25]. Only one study, a 2008 study of 276 PWTB in southern India, found no gender difference in social stigma, and reported that males had higher perceived stigma compared to women [40].

Compared to men, female PWTB described more anticipated stigma and faced more pressure to keep their disease status secret, even from family members [34,36,41]. In interviews of 2,115 PWTB in Tiruvallur, women were more likely than men to describe feeling inhibited discussing their treatment with family members (21% of women vs 14% of men) [9]. Community respondents stated that women were more likely to hide their TB diagnosis due to greater fears of isolation and rejection [42]. In a 1997 study in Mumbai that consisted of sixteen in-depth interviews with PWTB attending a government clinic, the women described a desire to hide their disease status, as well as increased difficulties doing so due to closer monitoring of their movements compared to men [41]. As women are more likely than men to work as domestic workers, they are particularly vulnerable to dismissal if their employers learned of their TB status [41]. Women even reported hiding their disease status from their husbands and fearing that their disease status stigmatized their children [34]. In a 2008 study, female sex was significantly associated with diagnostic delay, partially due to disease-related stigma exacerbated by gender [34].

A study in Kerala that presented community members with vignettes focusing on PWTB found that stigma was highest among women receiving female vignettes (p < 0.0001) [43]. Moreover, perceived stigma as it pertains to marital prospects disproportionately affects women. Female PWTB and their family members regularly described fearing reduced marriage prospects due to TB stigma [13,17,22,36,41]. Stigma can also have a negative effect on the matrimonial prospects of unmarried siblings and children of women with TB [17,19,22]. Community respondents stated that men - but not women - would be able to get married after recovering from TB [13]. Community members in Maharashtra, particularly men, disclosed that they did not want their sons to marry women with a history of TB [42]. Indeed, in a 2021 study in Tamil Nadu, five household members of PWTB divulged hiding either their wife’s or daughter’s TB diagnosis so that their daughters’ marriage prospects would not be affected [19].

The majority of studies found that women experienced more enacted stigma due to their TB status compared to men [5,8,9,15,17,20,36,41,44]. It is unclear whether there are gender differences in internalized stigma.

Not only do women experience greater stigma than men, they also receive less support than men. Two studies found that married female PWTB received less family support than married male or unmarried female PWTB [41,44]. While men generally reported their main concerns as being loss of wages and an inability to work, women primarily mentioned concerns regarding rejection from their husbands and in-laws [41]. Among PWTB in southern India, 69% of men but only 31% of women received support from their in-laws [40]. Women also described leaving treatment due to the pressure of household activities. In one study, community members stated that women would be obliged to support their husbands if they developed TB, but men would not be obliged to support their wives [42].

### Impacts of stigma

Stigma is connected to mental health, with perceived stigma significantly associated with depression (AOR 3.2, 95% CI 1.1-5.3) and anxiety (AOR 2.2, 95% CI 1.1-6.3) in those with MDR-TB [37].

A 2010 study conducted 21 in-depth interviews with PWTB and 13 in-depth interviews with community health workers in Gujarat Province [17]. They found that PWTB reported fearing the disclosure of their disease more than they feared TB itself [17]. This fear of disclosure can have a direct impact on seeking medical care, reducing treatment adherence and completion. In a qualitative study from Kolkata, PWTB admitted delaying diagnosis for months due to fear of TB stigma and reflected that they may have acquired the disease from other individuals who had also delayed medical care due to stigma [28].

The desire to hide disease status has a direct impact on medical treatment. Many Indians receive directly observed therapy administered by community health workers and family members, and some PWTB described having their status disclosed by community members who had seen them receiving treatment [13,17]. Healthcare workers reported that PWTB asked them to stop visiting their homes for treatment or declined to visit treatment centers due to fear of disclosure [17,36,45]. A study in Thiruvallur interviewed 612 PWTB, and found that 6.7% of PWTB provided false names and addresses to avoid their disease status being exposed [16]. Elsewhere, healthcare workers described cases where PWTB refused to take medication in front of family members or in-laws, including one case where the person subsequently died [17]. Stigma can have effects even when not enacted, with these instances of anticipated stigma having direct impacts on PWTBs’ health and well-being.

The role of stigma on treatment adherence has been quantified. In a study in West Bengal of 145 people who defaulted from TB treatment, 51 (35%) were categorized as being in a ‘low stigma’ group and 94 (65%) in a ‘high stigma’ group. Those in the low stigma group were 8.59 times more likely to disengage from therapy later (more than 30 days after starting treatment, compared to 0–30 days after starting treatment) than those in the high stigma group (p = 0.001), suggesting that reducing stigma could improve adherence to treatment [10]. A 2024 study in Jharkhand and a 2016 study in Gujarat both found that stigma was a major barrier to treatment adherence [35,46]. Another study found that perceived discrimination was a predictor of unsuccessful treatment (AOR 2.61, 95% CI 1.04–7.84) [28]. A study in eastern India found that PWTB who reported high levels of stigma had 20.27 times higher odds of nonadherence [11].

A 2022 study in Sikkim found that increased stigma is significantly associated with depression, which then predicts lower adherence to treatment [39]. Another study found that PWTB who experienced stigma were 15-fold more likely to have depression than PWTB who did not have experience stigma [47]. Similarly, a study focusing on persons with drug-resistant TB found that those who experienced stigma were three times more likely to have depression than those without stigma [48]. A study in Gujarat found that PWTB who experienced high levels of stigma were 2.3 times more likely to experience depression (95% CI: 1.1-2.3), and this trend also extended to household contacts, with those experiencing high perceived stigma being 1.8 times more likely to experience depression (95% CI: 1.1-2.3) [49].

### Knowledge and interventions to mitigate stigma

Despite the suggestion that education and awareness could improve stigma [16], studies examining the role of knowledge on stigmatizing attitudes and behaviors have had mixed results. One study found that training was able to decrease stigmatizing behavior among physicians [32], while another study found that limited knowledge about TB was significantly associated with stigmatizing attitudes (OR 14.99, 95% CI 6.98–32.1) [23]. However, a study of 3,823 participants in 30 districts using semi-structured questionnaires found that stigmatizing and discriminating attitudes were independent of respondents’ knowledge of TB [29]. In a 2024 study, PWTB were more likely to experience stigma if they themselves had reduced knowledge of TB (OR 3, 95% CI 1.005–8.95) [50]. A 2023 study found that PWTB with lower TB knowledge were more likely to experience stigma, and those who experienced stigma were then less likely to visit the TB clinic (p < 0.05) [25].

The 2021 Tamil Nadu and Puducherry study specifically focused on potential interventions to reduce community stigma and included interviews and focus groups with PWTB, household members, and key informants (such as program managers, government officials, and teachers) [19]. Participants’ suggestions to reduce community stigma included involving the entertainment industry; using social media to increase awareness; educating schoolchildren about TB; and instituting community interventions such as puppet shows or festivals. Participants also suggested counseling and support groups as ways to reduce internalized stigma among PWTB and their family members [19]. Table 2 shows potential interventions to reduce stigma or minimize its effects.

**Table 2:** Potential interventions for each type of stigma.

Type of stigma	Potential interventions
Anticipated	Support groups for PWTB and their families [19] Options to receive care at a secondary location or from non-uniformed healthcare workers
Perceived	Education programs for children in schools [19] Community groups to raise awareness on TB transmission and treatability [19] Training programs for physicians and other healthcare workers [32,55]
Enacted	Workplace protections for PWTB Community education programs, including social media [19] Celebrity-led campaigns on TB stigma [19] TB clubs [60]
Internalized	Support groups for PWTB and their families [19] Counseling and mental health support [58] TB clubs [60]

## Discussion

Our review of the literature found that stigma persists, is widely prevalent, and exacts heavy social costs upon PWTB. Understanding and countering all kinds of stigma –anticipated, perceived, enacted, and internalized—is essential. One key finding was that enacted stigma was less prevalent than anticipated and perceived stigma. This finding was consistent with research in stigma related to other diseases, such as HIV and podoconiosis [51–53], and suggests that interventions may need to address the specific type of stigma. Moreover, it is important to consider that enacted stigma may have been reduced due to PWTB hiding or not disclosing their disease status.

This review also found that women suffer stigma disproportionately, and interventions to reduce stigma should focus on these additional challenges faced by female PWTB. Women in India often experience social and gender inequities, and the added burden of TB-related stigma can further exacerbate these inequities. Stigma not only affects the mental health of PWTB and promotes ostracism, it also negatively affects healthcare providers’ ability to engage PWTB in care, particularly when some healthcare providers are themselves sources of stigma. Crucially, TB stigma can contribute to diagnostic delays and disengagement with care. TB stigma remains an important obstacle to ending the TB epidemic in India.

There is a paucity of research on interventions to reduce stigma [54]. Understanding the cultural underpinnings of TB stigma is critical to designing effective strategies to curb stigma. Additional quantitative and qualitative studies are needed to address the disparate experiences across geographic regions and cultural groups, and to understand which interventions are successful in reducing each type of TB-related stigma. In addition to devising interventions for community members and PWTB, addressing TB stigma among healthcare workers and preventing stigmatizing behavior during treatment is imperative. Given the mixed results on whether education reduces stigma, more innovation is needed in ways of reducing stigmatizing behaviors from healthcare providers. Studies focusing on other diseases, such as HIV, have found success in developing educational interventions for healthcare workers, particularly when multiple sessions are offered [55]. It is critical to incorporate the perspectives of PWTB in designing interventions to reduce stigma, as well as the expertise of community leaders. Moreover, stigma should be considered within the context of broader structural and social inequities, particularly within the healthcare system.

In addition, it is important to consider that stigma often arises from the fear of getting the disease. Education and awareness campaigns for household contacts and community members should focus on when isolation may be required and when it is no longer necessary (e.g., after adequate treatment and sputum smear conversion). Providing this information could help reduce stigma towards PWTB, while at the same time assuaging concerns about disease transmission.

Studying successful interventions from other contexts may be necessary. In Pakistan, storytelling through spoken word and photography have been used to mitigate both perceived stigma among community members as well as anticipated and internalized stigma among PWTB [56,57]. In Peru, psychological counseling was used to reduce internalized stigma in PWTB, particularly among women [58]. Enacted stigma from family members has been mitigated through community nurses who counseled family members to end ostracizing behavior [59]. However, interventions in India are likely to look different than those in other settings, due to India’s specific social and demographic context, as well as the unique gender dynamics. Interventions may also need to be adapted for different geographic contexts within India.

Further research is necessary on the relationship between knowledge and stigma surrounding TB, with the possibility that some educational interventions may be more effective than others in reducing stigma [29]. Another possible avenue for exploration is to foster TB clubs, which can provide a community for PWTB that reduces isolation and improves adherence. TB clubs also help spread awareness of treatment for TB [60]. TB clubs could decrease internalized and perceived stigma among participants, while also potentially decreasing enacted stigma by educating community members, and have already seen some success in India. However, TB clubs may not work in some contexts, as attending a club may itself increase stigma for PWTB. TB clubs should carefully consider their meeting location and other publicly available information in order to avoid increasing stigma.

We recommend that interventions focus especially on women, who are disproportionately affected by the stigma of TB and are offered less support by the community. Consideration needs to be given to the location and scheduling of these groups in a way that facilitates membership, particularly among women, and does not lead to additional stigma by members of the community.

In addition, future interventions should build on the work being done by TB advocates in India. Groups such as the Global Coalition of TB Advocates (GCTA) have developed training materials for incorporating social interventions alongside medical interventions. For instance, GCTA has developed a manual for a one-day training for healthcare workers and community members, with a focus on ending TB stigma. Programs such as this one may improve participant knowledge and develop strategies for reducing stigma [61].

This study has several limitations to acknowledge. First, India is a geographically, linguistically, and culturally diverse country, with a population of 1.4 billion. The lived experiences of PWTB may be different in different contexts and settings and may not be expressed in the studies included in this narrative review. This review also only included studies published in English. Moreover, our search strategy was not intended to be exhaustive, and not all studies that concern TB stigma in India were included. In general, studies focusing on PWTB used enrollment in a public sector treatment center as their criterion for inclusion. Individuals treated in the private sector and those who forewent therapy were generally not included in this study. Such PWTBs may have experienced different levels of perceived and enacted stigma. Many of the studies cited in this review used qualitative methods and open-ended interviewing, which can be harder to quantify and compare between studies. However, the use of EMIC and other validated methods allowed for stigma to be quantified and allows comparison between different contexts.

Even as we invest heavily in improving the biomedical dimension of TB care, we must address the pervasive TB stigma that haunts millions of PWTB. Further action is also crucial to improve the quality of life of PWTB during and after their treatment, particularly from the psychological perspective. ‘The biggest disease today,’ Mother Teresa said, ‘is not leprosy or tuberculosis, but rather the feeling of being unwanted, uncared for and deserted by everybody.’

## Supporting information

S1 Text The MeSH search terms used in this study. (DOCX)
