# Supplementary material for: TB stigma in India: A narrative review of types of stigma, gender differences, and potential interventions
Source: PLOS Glob Public Health. 2025 Sep 22;5(9):e0005109. doi: 10.1371/journal.pgph.0005109 (PMC12453202; doi:10.1371/journal.pgph.0005109)
Supplement: S1 Text — (DOCX) [file pgph.0005109.s001.docx]

**Supplemental Material**

MeSH Search Terms

("India"[Mesh] OR India OR "Indian Subcontinent*" OR "indian sub continent*" OR "south* Indian" OR "Arunachal Pradesh" OR Itanagar OR Assam OR Dhuburi OR Dibrugarh OR Dispur OR Guwahati OR Jorhat OR Nagaon OR Sibsagar OR Silchar OR Tezpur OR Tinsukia OR Bihar OR Ara OR Baruni OR Begusarai OR Bettiah OR Bhagalpur OR "Bihar Sharif" OR "Bodh Gaya" OR Buxar OR Chapra OR Darbhanga OR Dehri OR "Dinapur Nizamat" OR Gaya OR Hajipur OR Jamalpur OR Katihar OR Madhubani OR Motihari OR Munger OR Muzaffarpur OR Patna OR Purnia OR Pusa OR Saharsa OR Samastipur OR Sasaram OR Sitamarhi OR Siwan OR Chandigarh OR Chandigarh OR Chhattisgarh OR Ambikapur OR Bhilai OR Bilaspur OR "Dhamtari Durg" OR Jagdalpur OR Raipur OR Rajnandgaon OR Dadra OR "Nagar Haveli" OR Silvassa OR Diu OR Daman OR Delhi OR Madgaon OR Panaji OR Gujarat OR Ahmadabad OR Amreli OR Bharuch OR Bhavnagar OR Bhuj OR Dwarka OR Gandhinagar OR Godhra OR Jamnagar OR Junagadh OR Kandla OR Khambhat OR Kheda OR Mahesana OR Morvi OR Nadiad OR Navsari OR Okha OR Palanpur OR Patan OR Porbandar OR Rajkot OR Surat OR Surendranagar OR Valsad OR Veraval OR Haryana OR Ambala OR Bhiwani OR Chandigarh OR Faridabad OR "Firozpur Jhirka" OR Gurgaon OR Hansi OR Hisar OR Jind OR Kaithal OR Karnal OR Kurukshetra OR Panipat OR Pehowa OR Rewari OR Rohtak OR Sirsa OR Sonipat OR "Himachal Pradesh" OR Bilaspur OR Chamba OR (Dalhousie NOT (Halifax or Canada or England)) OR Dharmshala OR Hamirpur OR Kangra OR Kullu OR Mandi OR Nahan OR Shimla OR Jammu OR Kashmir OR Anantnag OR Baramula OR Doda OR Gulmarg OR Jammu OR Kathua OR Rajauri OR Srinagar OR Udhampur OR Jharkhand OR Bokaro OR Chaibasa OR Deoghar OR Dhanbad OR Dumka OR Giridih OR Hazaribag OR Jamshedpur OR Jharia OR Rajmahal OR Ranchi OR Saraikela OR Karnataka OR Badami OR Ballari OR Bangalore OR Belgavi OR Bhadravati OR Bidar OR Chikkamagaluru OR Chitradurga OR Davangere OR Halebid OR Hubballi Dharwad OR Kalaburagi OR Kolar OR Madikeri OR Mandya OR Mangaluru OR Mysuru OR Raichur OR Shivamogga OR Shravanabelagola OR Shrirangapattana OR Tumkuru OR Kerala OR Alappuzha OR Badagara OR Idukki OR Kannur OR Kochi OR Kollam OR Kottayam OR Kozhikode OR Mattancheri OR Palakkad OR Thalassery OR hiruvananthapuram OR Thrissur OR "Madhya Pradesh" OR Balaghat OR Barwani OR Betul OR Bharhut OR Bhind OR Bhojpur OR Bhopal OR Burhanpur OR Chhatarpur OR Chhindwara OR Damoh OR Datia OR Dewas OR Dhar OR Guna OR Gwalior OR Hoshangabad OR Indore OR Itarsi OR Jabalpur OR Jhabua OR Khajuraho OR Khandwa OR Khargon OR Maheshwar OR Mandla OR Mandsaur OR Mhow OR Morena OR Murwara OR Narsimhapur OR Narsinghgarh OR Narwar OR Neemuch OR Nowgong OR Orchha OR Panna OR Raisen OR Rajgarh OR Ratlam OR Rewa OR Sagar OR Sarangpu OR Satna OR Sehore OR Seoni OR Shahdol OR Shajapur OR Sheopur OR Shivpuri OR Ujjain OR Vidisha OR Maharashtra OR Ahmadnagar OR Akola OR Amravati OR Aurangabad OR Bhandara OR Bhusawal OR Buldana OR Chandrapur OR Daulatabad OR Dhule OR Jalgaon OR Kalyan OR Karli OR Kolhapur OR Mahabaleshwar OR Malegaon OR Matheran OR Mumbai OR Nagpur OR Nanded OR Nashik OR Osmanabad OR Pandharpur OR Parbhani OR Pune OR Ratnagiri OR Sangli OR Satara OR Sevagram OR Solapur OR Ulhasnagar OR Vasai Virar OR Wardha OR Yavatmal OR Manipur OR Imphal OR Meghalaya OR Cherrapunji OR Shillong OR Mizoram OR Aizawl OR Lunglei OR Nagaland OR Kohima OR Phek OR Wokha OR Zunheboto OR Odisha OR Balangir OR Baleshwar OR Baripada OR Bhubaneshwar OR Brahmapur OR Cuttack OR Dhenkanal OR Keonjhar OR Konark OR Koraput OR Paradip OR Phulabani OR Puri OR Sambalpur OR Udayagiri OR Puducherry OR Karaikal OR Mahe OR Yanam OR Punjab OR Amritsar OR Batala OR Chandigarh OR Faridkot OR Firozpur OR Gurdaspur OR Hoshiarpur OR Jalandhar OR Kapurthala OR Ludhiana OR Nabha OR Patiala OR Rupnagar OR Sangrur OR Rajasthan OR Ajmer OR Alwar OR Amer OR Barmer OR Beawar OR Bharatpur OR Bhilwara OR Bikaner OR Bundi OR Chittaurgarh OR Churu OR Dhaulpur OR Dungarpur OR Ganganagar OR Hanumangarh OR Jaipur OR Jaisalmer OR Jalor OR Jhalawar OR Jhunjhunu OR Jodhpur OR Kishangarh OR Kota OR Merta OR Nagaur OR Nathdwara OR Pali OR Phalodi OR Pushkar OR "Sawai Madhopur" OR Shahpura OR Sikar OR Sirohi OR Tonk OR Udaipur OR Sikkim OR Gangtok OR Gyalsing OR Lachung OR Mangan OR "Tamil Nadu" OR Arcot OR Chengalpattu OR Chennai OR Chidambaram OR Coimbatore OR Cuddalore OR Dharmapuri OR Dindigul OR Kanchipuram OR Kanniyakumari OR Kodaikanal OR Kumbakonam OR Madurai OR Mamallapuram OR Nagappattinam OR Nagercoil OR Palayankottai OR Pudukkottai OR Rajapalaiyam OR Ramanathapuram OR Thanjavur OR Tiruchchirappalli OR Tirunelveli OR Tiruppur OR Tuticorin OR Udhagamandalam OR Vellore OR Telangana OR Hyderabad OR Karimnagar OR Khammam OR Mahbubnagar OR Nizamabad OR Sangareddi OR Warangal OR Tripura OR Agartala OR "Uttar Pradesh" OR Agra OR Aligarh OR Allahabad OR Amroha OR Ayodhya OR Azamgarh OR Bahraich OR Ballia OR Banda OR "Bara Banki" OR Bareilly OR Basti OR Bijnor OR Bithur OR Budaun OR Bulandshahr OR Deoria OR Etah OR Etawah OR Faizabad OR "Farrukhabad cum Fatehgarh" OR Fatehpur OR Fatehpur Sikri OR Ghaziabad OR Ghazipur OR Gonda OR Gorakhpur OR Hamirpur OR Hardoi OR Hathras OR Jalaun OR Jaunpur OR Jhansi OR Kannauj OR Kanpur OR Lakhimpur OR Lalitpur OR Lucknow OR Mainpuri OR Mathura OR Meerut OR Mirzapur Vindhyachal OR Moradabad OR Muzaffarnagar OR Partapgarh OR Pilibhit OR "Rae Bareli " OR Rampur OR Saharanpur OR Sambhal OR Shahjahanpur OR Sitapur OR Sultanpur OR Tehri OR Varanasi OR Uttarakhand OR Almora OR "Dehra Dun" OR Haridwar OR Mussoorie OR Nainital OR Pithoragarh OR West Bengal OR Alipore OR "Alipur Duar" OR Asansol OR Baharampur OR Bally OR Balurghat OR Bankura OR Baranagar OR Barasat OR Barrackpore OR Basirhat OR Bhatpara OR Bishnupur OR "Budge Budge" OR Burdwan OR Chandernagore OR Darjiling OR "Diamond Harbour" OR "Dum Dum" OR Durgapur OR Halisahar OR Haora OR Hugli OR "Ingraj Bazar" OR Jalpaiguri OR Kalimpong OR Kamarhati OR Kanchrapara OR Kharagpur OR Koch Bihar OR Kolkata OR Krishnanagar OR Malda OR Midnapore OR Murshidabad OR Navadwip OR Palashi OR Panihati OR Purulia OR Raiganj OR Santipur OR Shantiniketan OR Shrirampur OR Siliguri OR Siuri OR Tamluk OR Titagarh) AND ("Tuberculosis"[Mesh] OR "Tuberculosis, Pulmonary"[Mesh] OR "Latent Tuberculosis"[Mesh] OR "Extensively Drug-Resistant Tuberculosis"[Mesh] OR "Tuberculosis, Central Nervous System"[Mesh] OR "Tuberculosis, Multidrug-Resistant"[Mesh] OR "Mycobacterium tuberculosis"[Mesh] OR "Antitubercular agents" [Mesh] OR "Tuberculin test"[Mesh] OR "Tuberculosis Vaccines"[Mesh] OR "Tuberculosis, Urogenital"[Mesh] OR "Tuberculosis, Splenic"[Mesh] OR "Tuberculosis, Spinal"[Mesh] OR "Tuberculosis, Renal"[Mesh] OR "Tuberculosis, Pleural"[Mesh] OR "Tuberculosis, Osteoarticular"[Mesh] OR "Tuberculosis, Oral"[Mesh] OR "Tuberculosis, Ocular"[Mesh] OR "Tuberculosis, Miliary"[Mesh] OR "Tuberculosis, Meningeal"[Mesh] OR "Tuberculosis, Male Genital"[Mesh] OR "Tuberculosis, Lymph Node"[Mesh] OR "Tuberculosis, Laryngeal"[Mesh] OR "Tuberculosis, Hepatic"[Mesh] OR "Tuberculosis, Gastrointestinal"[Mesh] OR "Tuberculosis, Female Genital"[Mesh] OR "Tuberculosis, Endocrine"[Mesh] OR "Tuberculosis, Cutaneous"[Mesh] OR "Tuberculosis, Cardiovascular"[Mesh] OR "Tuberculosis, Extrapulmonary"[Mesh] OR "Drug resistant TB" OR "Tuberculosis stigma" OR "TB" OR "Multidrug-resistant tuberculosis" OR "MDR-TB" OR "MDR TB" OR "Pulmonary tuberculosis" OR "Tuberculosis" OR "Drug resistant tuberculosis" OR "Drug-resistant tuberculosis") AND ("Cost of Illness"[Mesh] OR "Health Knowledge, Attitudes, Practice"[Mesh] OR "Prejudice"[Mesh] OR "Social Discrimination"[Mesh] OR "Social Isolation"[Mesh] OR "Social Stigma"[Mesh] OR "Social Problems"[Mesh] OR "Social Support"[Mesh] OR "Socioeconomic Factors"[Mesh] OR "Stereotyping"[Mesh] OR "Patient acceptance of health care"[Mesh] OR "Treatment Adherence and Compliance"[Mesh] OR "Attitude to health"[Mesh] OR "Stress, psychological"[Mesh] OR "Cost of illness" OR "Stigma" OR "Social stigma" OR "Social stigmas" OR "Internalized stigma" OR "Shame" OR "Prejudices" OR "Depression" OR "Anxiety" OR "Mental Health" OR "Discrimination" OR "Indirect costs" OR "Costs" OR "Cost" OR "Coping" OR "Stigma Reduction Interventions" OR "Stigma Reduction Intervention" OR "Social impact" OR "Quality of life" OR "Illness Cost" OR "Illness Costs" OR "Burden Of Disease" OR "Burden Of Diseases" OR "Cost of Sickness" OR "Sickness Costs" OR "Sickness Cost" OR "Burden of Illness" OR "Illness Burden" OR "Illness Burdens" OR "Cost of Disease" OR "Costs of Disease" OR "Disease Cost" OR "Cost, Disease" OR "Disease Costs" OR "Disease Burden" OR "Burden, Disease" OR "Disease Burdens" OR "Social norms" OR "Social norm" OR "Social mores")
